# Supplementary material for: The role of structural pleiotropy and regulatory evolution in the retention of heteromers of paralogs
Source: eLife. 2019 Aug 27;8:e46754. doi: 10.7554/eLife.46754 (PMC6711710; doi:10.7554/eLife.46754)
Supplement: Supplementary file 1. [file elife-46754-supp1.docx]

**Supplementary material of Marchant et al.**

**Table of contents:**

**Page**

Supplementary text 2

Comparison of PCA results with previous studies 2

Table S1: Homomer data 2

Table S2: Table S1 Headers 2

Table S3: Summary results per pair of paralogs 2

Table S4: Table S3 Headers 2

Table S5: Description of RNAseq samples 2

Table S6: Number of RNAseq reads per gene 2

Table S7: Results from GLM on protein interactions versus expression levels 2

Table S8: Counts of mutation types in the simulations of protein evolution 3

Table S9: List of paralog pairs 3

Table S10: Table S9 Headers 3

Table S11: Culture media used in this study 3

Table S12: Oligonucleotides used in this study 3

Table S13: PDB structures used in this study 3

Table S14: Summary of results for pairs of human paralogs 3

Table S15: Table S14 headers 3

###### Supplementary text

###### Comparison of PCA results with previous studies

We performed a screen using PCA based on the DHFR [(Tarassov et al. 2008)](https://paperpile.com/c/sNkzgD/221Om) to test for PPIs among SSDs and WGDs in *Saccharomyces cerevisiae*, specifically testing for self-interactions (HMs) and interactions between paralogs (HETs). The yeast DHFR PCA detects direct and near direct interactions without disturbing endogenous regulation, giving insight into the role of transcriptional regulation in the evolution of PPIs [(Tarassov et al. 2008; Rochette et al. 2014; Barshir et al. 2018; Gagnon-Arsenault et al. 2013)](https://paperpile.com/c/sNkzgD/221Om+pkorJ+9dnoj+mwVtY). PCA is one of the standard binary methods used to measure direct and near-direct PPIs in yeast and mammalian cells [(Titeca et al. 2019)](https://paperpile.com/c/sNkzgD/LFTsR). PCA’s performance compares to other standard methods when proper controls and analyses are performed. It has been used successfully by our group and others in various contexts since its first application [(Schlecht et al. 2017; Celaj et al. 2017; Chrétien et al. 2018; Stynen et al. 2018; Lev, Volpe, and Ben-Aroya 2014)](https://paperpile.com/c/sNkzgD/PaSCa+9V8YU+J5mfr+eckqk+WuDXk).

In general, the PCA signal in our study strongly correlates with results from previous PCA experiments [(Stynen et al. 2018; Tarassov et al. 2008)](https://paperpile.com/c/sNkzgD/eckqk+221Om) and other publicly available data (Figure 2-figure supplement 1). Roughly 75% of the HMs and HETs detected in our PCA experiments were previously reported (Figure 2-figure supplement 2, Tables S3 and S4), suggesting that most of the HMs and HETs that can be detected with the available tools and in standard conditions have been discovered. While 76 HMs and 47 HETs reported in other studies were not detected in our PCA, our experiments detected 44 HMs and 19 HETs not previously reported (Tables S3 and S4).

###### Table S1: Homomer data

Homomers among proteins from singleton and duplicated genes: results from PCA experiments performed in this study and from published studies and databases.

###### Table S2: Table S1 Headers

Detailed description of Table S3 column headers.

###### Table S3: Summary results per pair of paralogs

Homomers and heteromers of paralogs: results from PCA experiments performed in this study and from published studies and databases.

###### Table S4: Table S3 Headers

Detailed description of Table S1 column headers.

###### Table S5: Description of RNAseq samples

Number of reads before and after cleaning for the RNAseq experiments in the DHFR PCA growth conditions.

###### Table S6: Number of RNAseq reads per gene

RNAseq reads mapped on the *S. cerevisiae* reference genome.

###### Table S7: Results from GLM on protein interactions versus expression levels

(**A**) Impact of expression levels (protein abundance and mRNA) and duplication on the detection of homomers. (**B**) Impact of the percentage of pairwise amino acid sequence identity and function similarity (GO, correlation of genetic interaction profiles, phenotype, localization and transcription factor) on the maintenance of interaction between paralogs after duplication of a HM (HM *versus* HM&HET). (**C**) Impact of the percentage of pairwise amino acid sequence identity and of the expression correlation profiles on the maintenance of interaction between paralogs after duplication of HMs (HM *versus* HM&HET).

For three models, we performed a GLM (binomial family) and then a Likelihood Ratio Test to estimate whether the factors significantly improved the model (assessed by the part of residual deviation explained by the factor). The factors tested and their data sources are described on the right side of each table. The following information is shown: estimates of the linear function and its standard error in brackets (Estimate (standard error) columns) for the intercept and factors tested, and proportion of residual deviance (Residual deviance columns). P-values for each test are indicated next to the estimated values and residual deviance: “*”(p < 0.05); “**” (p < 0.01); “***” (p < 0.001). The following information is provided from the Summ function of the jtools R package [(Long 2019)](https://paperpile.com/c/sNkzgD/w4ep): number of observations (N); Akaike Information Criterion (AIC); Bayesian Information Criterion (BIC) and pseudo regression (Pseudo R2).

###### Table S8: Counts of mutation types in the simulations of protein evolution

Counts of attempted and fixed mutations are shown for each of the complexes used in the simulations. Double mutants refer to steps in which both loci get a nonsynonymous substitution, single mutants refer to steps in which one of the loci gets a nonsynonymous substitution, and non-mutants refer to steps in which both loci get a synonymous substitution.

###### Table S9: List of paralog pairs

List of DHFR PCA strains from the DHFR collection of Tarassov et al., (2008) and from Diss et al., (2017). We also report if strain constructions were validated by PCR and if new DHFR PCA strains were constructed and validated for this study.

###### Table S10: Table S9 Headers

Detailed description of Table S9 column headers.

###### Table S11: Culture media used in this study

Description of media composition.

###### Table S12: Oligonucleotides used in this study

List of oligonucleotides used for construction and verification of DHFR PCA yeast strains and Gateway destination plasmids.

###### Table S13: PDB structures used in this study

List of PDB structures and biological assemblies used for the analyses of the conservation of interface sequences and the simulations of protein complex evolution. Proteins matched to the chains in that particular structure along with the counts of total residues and interface residues are shown.

###### Table S14: Summary of results for pairs of human paralogs

Interaction of homomers and heteromers of human paralogs: data from the merged BioGRID-IntAct dataset and the PDB.

###### Table S15: Table S14 headers

Detailed description of Table S14 column headers.

**References**

[Barshir, Ruth, Idan Hekselman, Netta Shemesh, Moran Sharon, Lena Novack, and Esti Yeger-Lotem. 2018. “Role of Duplicate Genes in Determining the Tissue-Selectivity of Hereditary Diseases.” *PLoS Genetics* 14 (5): e1007327.](http://paperpile.com/b/sNkzgD/9dnoj)

[Berman, H. M., J. Westbrook, Z. Feng, G. Gilliland, T. N. Bhat, H. Weissig, I. N. Shindyalov, and P. E. Bourne. 2000. “The Protein Data Bank.” *Nucleic Acids Research* 28 (1): 235–42.](http://paperpile.com/b/sNkzgD/WQbPH)

[Celaj, Albi, Ulrich Schlecht, Justin D. Smith, Weihong Xu, Sundari Suresh, Molly Miranda, Ana Maria Aparicio, et al. 2017. “Quantitative Analysis of Protein Interaction Network Dynamics in Yeast.” *Molecular Systems Biology* 13 (7): 934.](http://paperpile.com/b/sNkzgD/9V8YU)

[Chatr-Aryamontri, Andrew, Bobby-Joe Breitkreutz, Sven Heinicke, Lorrie Boucher, Andrew Winter, Chris Stark, Julie Nixon, et al. 2013. “The BioGRID Interaction Database: 2013 Update.” *Nucleic Acids Research* 41 (D1): D816–23.](http://paperpile.com/b/sNkzgD/y3emE)

[Chatr-Aryamontri, Andrew, Rose Oughtred, Lorrie Boucher, Jennifer Rust, Christie Chang, Nadine K. Kolas, Lara O’Donnell, et al. 2017. “The BioGRID Interaction Database: 2017 Update.” *Nucleic Acids Research* 45 (D1): D369–79.](http://paperpile.com/b/sNkzgD/MW8co)

[Chrétien, Andrée-Ève, Isabelle Gagnon-Arsenault, Alexandre K. Dubé, Xavier Barbeau, Philippe C. Després, Claudine Lamothe, Anne-Marie Dion-Côté, Patrick Lagüe, and Christian R. Landry. 2018. “Extended Linkers Improve the Detection of Protein-Protein Interactions (PPIs) by Dihydrofolate Reductase Protein-Fragment Complementation Assay (DHFR PCA) in Living Cells.” *Molecular & Cellular Proteomics: MCP* 17 (2): 373–83.](http://paperpile.com/b/sNkzgD/J5mfr)

[Gagnon-Arsenault, Isabelle, François-Christophe Marois Blanchet, Samuel Rochette, Guillaume Diss, Alexandre K. Dubé, and Christian R. Landry. 2013. “Transcriptional Divergence Plays a Role in the Rewiring of Protein Interaction Networks after Gene Duplication.” *Journal of Proteomics*, Special Issue: From protein structures to clinical applications, 81 (April): 112–25.](http://paperpile.com/b/sNkzgD/mwVtY)

[Gasch, Audrey P., Feiqiao Brian Yu, James Hose, Leah E. Escalante, Mike Place, Rhonda Bacher, Jad Kanbar, et al. 2017. “Single-Cell RNA Sequencing Reveals Intrinsic and Extrinsic Regulatory Heterogeneity in Yeast Responding to Stress.” *PLoS Biology* 15 (12): e2004050.](http://paperpile.com/b/sNkzgD/5MsY)

[Ihmels, Jan, Sven Bergmann, and Naama Barkai. 2004. “Defining Transcription Modules Using Large-Scale Gene Expression Data.” *Bioinformatics*  20 (13): 1993–2003.](http://paperpile.com/b/sNkzgD/H0W9V)

[Kim, Yeonsoo, Jong Pil Jung, Chan-Gi Pack, and Won-Ki Huh. 2019. “Global Analysis of Protein Homomerization in Saccharomyces Cerevisiae.” *Genome Research* 29 (1): 135–45.](http://paperpile.com/b/sNkzgD/GGKMI)

[Lev, Ifat, Marina Volpe, and Shay Ben-Aroya. 2014. “Identification of Genes Important for the Physical Interaction between Protein Pairs through Reverse PCA (rPCA).” *Current Protocols in Cell Biology / Editorial Board, Juan S. Bonifacino ... [et Al.]* 64 (September): 17.15.1–11.](http://paperpile.com/b/sNkzgD/WuDXk)

[Lex, Alexander, Nils Gehlenborg, Hendrik Strobelt, Romain Vuillemot, and Hanspeter Pfister. 2014. “UpSet: Visualization of Intersecting Sets.” *IEEE Transactions on Visualization and Computer Graphics* 20 (12): 1983–92.](http://paperpile.com/b/sNkzgD/cWp3k)

[Long, Jacob A. 2019. “Analysis and Presentation of Social Scientific Data [R Package Jtools Version 2.0.1].”](http://paperpile.com/b/sNkzgD/w4ep) <https://cran.r-project.org/web/packages/jtools/index.html>[.](http://paperpile.com/b/sNkzgD/w4ep)

[Rochette, Samuel, Isabelle Gagnon-Arsenault, Guillaume Diss, and Christian R. Landry. 2014. “Modulation of the Yeast Protein Interactome in Response to DNA Damage.” *Journal of Proteomics*, Special Issue: Can Proteomics Fill the Gap Between Genomics and Phenotypes?, 100 (April): 25–36.](http://paperpile.com/b/sNkzgD/pkorJ)

[Schlecht, Ulrich, Zhimin Liu, Jamie R. Blundell, Robert P. St Onge, and Sasha F. Levy. 2017. “A Scalable Double-Barcode Sequencing Platform for Characterization of Dynamic Protein-Protein Interactions.” *Nature Communications* 8 (May): 15586.](http://paperpile.com/b/sNkzgD/PaSCa)

[Stynen, Bram, Diala Abd-Rabbo, Jacqueline Kowarzyk, Leonor Miller-Fleming, Simran Kaur Aulakh, Philippe Garneau, Markus Ralser, and Stephen W. Michnick. 2018. “Changes of Cell Biochemical States Are Revealed in Protein Homomeric Complex Dynamics.” *Cell*, October. https://doi.org/](http://paperpile.com/b/sNkzgD/eckqk)[10.1016/j.cell.2018.09.050](http://dx.doi.org/10.1016/j.cell.2018.09.050)[.](http://paperpile.com/b/sNkzgD/eckqk)

[Tarassov, Kirill, Vincent Messier, Christian R. Landry, Stevo Radinovic, Mercedes M. Serna Molina, Igor Shames, Yelena Malitskaya, Jackie Vogel, Howard Bussey, and Stephen W. Michnick. 2008. “An in Vivo Map of the Yeast Protein Interactome.” *Science* 320 (5882): 1465–70.](http://paperpile.com/b/sNkzgD/221Om)

[Titeca, Kevin, Irma Lemmens, Jan Tavernier, and Sven Eyckerman. 2019. “Discovering Cellular Protein-Protein Interactions: Technological Strategies and Opportunities.” *Mass Spectrometry Reviews* 38 (1): 79–111.](http://paperpile.com/b/sNkzgD/LFTsR)
