# Supplementary material for: The role of structural pleiotropy and regulatory evolution in the retention of heteromers of paralogs
Source: eLife. 2019 Aug 27;8:e46754. doi: 10.7554/eLife.46754 (PMC6711710; doi:10.7554/eLife.46754)
Supplement: Transparent reporting form [file elife-46754-transrepform.docx]

***eLife’s* transparent reporting form**

We encourage authors to provide detailed information *within their submission* to facilitate the interpretation and replication of experiments. Authors can upload supporting documentation to indicate the use of appropriate reporting guidelines for health-related research (see [EQUATOR Network](http://www.equator-network.org/%20)), life science research (see the [BioSharing Information Resource](https://biosharing.org/)), or the [ARRIVE guidelines](http://www.plosbiology.org/article/info:doi/10.1371/journal.pbio.1000412) for reporting work involving animal research. Where applicable, authors should refer to any relevant reporting standards documents in this form.

If you have any questions, please consult our Journal Policies and/or contact us: [editorial@elifesciences.org](mailto:editorial@elifesciences.org).

**Sample-size estimation**

- You should state whether an appropriate sample size was computed when the study was being designed
- You should state the statistical method of sample size computation and any required assumptions
- If no explicit power analysis was used, you should describe how you decided what sample (replicate) size (number) to use

Please outline where this information can be found within the submission (e.g., sections or figure legends), or explain why this information doesn’t apply to your submission:

We used the maximum number of paralog pairs than we could study based on homomers and heteromers reported in databases and on experimental results. (section Material and Methods)

For the plasmid based PCA experiment:

The number of replicates is indicated in Methods section DHFR PCA experiments. The number of replicates was determined based on previous studies reporting similar experiments.

The number of protein structures used in the simulations (six) was not calculated with an explicit power analysis. The evaluation of the generality of patterns was only performed qualitatively.

Sample size estimation does not apply for the interface conservation analysis, as we used all available data on crystal structures of protein complexes including duplicated proteins.

**Replicates**

- You should report how often each experiment was performed
- You should include a definition of biological versus technical replication
- The data obtained should be provided and sufficient information should be provided to indicate the number of independent biological and/or technical replicates
- If you encountered any outliers, you should describe how these were handled
- Criteria for exclusion/inclusion of data should be clearly stated
- High-throughput sequence data should be uploaded before submission, with a private link for reviewers provided (these are available from both GEO and ArrayExpress)

Please outline where this information can be found within the submission (e.g., sections or figure legends), or explain why this information doesn’t apply to your submission:

Experimental PCA results of homomers and heteromers came from 286 different paralog pairs. Each interaction was tested in five to twenty replicates (different mating events from the same parental strains).

mRNA for RNAseq were extracted from three different biological replicates. RNAseq data could be found here: <https://www.ncbi.nlm.nih.gov/Traces/study/?acc=PRJNA494421>

For the plasmid based PCA experiment:

The experiment was performed once. We did not find outliers. We did not exclude data from our analysis. All replicates described in our work are biological replicates, i.e. different mating events from the same parental strains.

The number of replicates for the simulations was limited to 50 replicates with 200 steps each to keep the analyses computationally tractable while offering a good sampling of the sequence space. Neutral evolution simulations show that the used framework can achieve destabilization of the complexes in the simulated time frame.

Replication does not apply to the interface conservation analyses.

**Statistical reporting**

- Statistical analysis methods should be described and justified
- Raw data should be presented in figures whenever informative to do so (typically when N per group is less than 10)
- For each experiment, you should identify the statistical tests used, exact values of N, definitions of center, methods of multiple test correction, and dispersion and precision measures (e.g., mean, median, SD, SEM, confidence intervals; and, for the major substantive results, a measure of effect size (e.g., Pearson's r, Cohen's d)
- Report exact p-values wherever possible alongside the summary statistics and 95% confidence intervals. These should be reported for all key questions and not only when the p-value is less than 0.05.

Please outline where this information can be found within the submission (e.g., sections or figure legends), or explain why this information doesn’t apply to your submission:

P-values and the tests used for each comparison can be found in the corresponding legends of each figure or result text.

For the plasmid based PCA experiment:

The statistical tests are described in the Methods section 3.4. Analysis of DHFR PCA results.

(For large datasets, or papers with a very large number of statistical tests, you may upload a single table file with tests, Ns, etc., with reference to sections in the manuscript.)

**Group allocation**

- Indicate how samples were allocated into experimental groups (in the case of clinical studies, please specify allocation to treatment method); if randomization was used, please also state if restricted randomization was applied
- Indicate if masking was used during group allocation, data collection and/or data analysis

Please outline where this information can be found within the submission (e.g., sections or figure legends), or explain why this information doesn’t apply to your submission:

Proteins were grouped based on whether they formed only homomers or they formed both homomers and heteromers of paralogs. This distinction allowed us to evaluate which was the most represented, whether conservation of interfaces was associated to the formation of heteromers of paralogs and link with protein expression and gene functions.

For the plasmid based PCA experiment:

Not applicable, we did not allocate samples into experimental groups.

**Additional data files (“source data”)**

- We encourage you to upload relevant additional data files, such as numerical data that are represented as a graph in a figure, or as a summary table
- Where provided, these should be in the most useful format, and they can be uploaded as “Source data” files linked to a main figure or table
- Include model definition files including the full list of parameters used
- Include code used for data analysis (e.g., R, MatLab)
- Avoid stating that data files are “available upon request”

Please indicate the figures or tables for which source data files have been provided:

Source data and code used to plot figures are provided in supplementary tables and in the Github repository in the data folders of each analysis : https://github.com/landrylaboratory/Gene_duplication_2019
